# Dr. Joseph Ayre

**Published:** 1860-03

**Authors:** 


DR. JOSEPH AYRE, of Hull, died on the 15th of January, of this year, of disease of the bladder, having attained the ripe old age of seventy-nine. He was an indefatigable worker, and a frequent contributor to various medical journals, his last production being a memoir—read in 1857 before the Academy of Sciences of Paris—on the best mode of curing cholera, entered in competition with a hundred and fifty other essays for the Briant prize.

The committee considered his treatise the best that was presented, but did not award the prize to it, as the collection of facts was not large enough to entitle his treatment to be considered as a specific.

Dr. Ayre published, in 1818, a work "on the Disorders of the Liver," and in 1826, a "Treatise on Dropsy," both of which were translated into German.

